# Bitpacking techniques for indexing genomes: I. Hash tables

**DOI:** 10.1186/s13015-016-0069-5

**Published:** 2016-04-18

**Authors:** Thomas D. Wu

**Affiliations:** Department of Bioinformatics and Computational Biology, Genentech, Inc., 1 DNA Way, 94080, South San Francisco, CA 94080 USA

**Keywords:** Hash table, Sequence alignment, Genomics, Data compression

## Abstract

**Background:**

Hash tables constitute a widely used data structure for indexing genomes that provides a list of genomic positions for each possible oligomer of a given size. The offset array in a hash table grows exponentially with the oligomer size and precludes the use of larger oligomers that could facilitate rapid alignment of sequences to a genome.

**Results:**

We propose to compress the offset array using vectorized bitpacking. We introduce an algorithm and data structure called BP64-columnar that achieves fast random access in arrays of monotonically nondecreasing integers. Experimental results based on hash tables for the fly, chicken, and human genomes show that BP64-columnar is 3 to 4 times faster than publicly available implementations of universal coding schemes, such as Elias gamma, Elias delta, and Fibonacci compression. Furthermore, among vectorized bitpacking schemes, our BP64-columnar format yields retrieval times that are faster than the fastest known bitpacking format by a factor of 3 for retrieving a single value, and a factor of 2 for retrieving two adjacent values.

**Conclusions:**

Our BP64-columnar scheme enables compression of genomic hash tables with fast retrieval. It also has potential applications to other domains requiring differential coding with random access.

**Electronic supplementary material:**

The online version of this article (doi:10.1186/s13015-016-0069-5) contains supplementary material, which is available to authorized users.

## Background

In bioinformatics applications, genomes are generally represented not as a linear string of nucleotides, but as indexed data structures that can facilitate various analyses, such as the rapid alignment of query reads. With the advent of high-throughput sequencing and the generation of unprecedented volumes of read data [1], speed has become paramount in genomic alignment. One major indexing method of preprocessing genomes for efficient alignment is a hash table. A hash table represents a genome sequence as multiple lists of genomic positions, one list for each possible *k*-mer, or oligomer of some preselected length *k*.

Hash tables are used by various programs, including blast [2], patternhunter [3], shrimp [4], blat [5], NextGenMap [6], and gmap [7], to identify short oligomer (or seed) matches between a read and the genome. These seeds can then be combined or extended to obtain a more complete alignment. Hash tables are particularly useful for aligning reads that include multiple mismatches or indels relative to a genome. Hash tables are also useful for applications where ambiguity arises in the nucleotide content at a given position, such as with single nucleotide polymorphisms, or SNPs, since a hash table can map two different oligomers onto the same position.

**Fig. 1:**
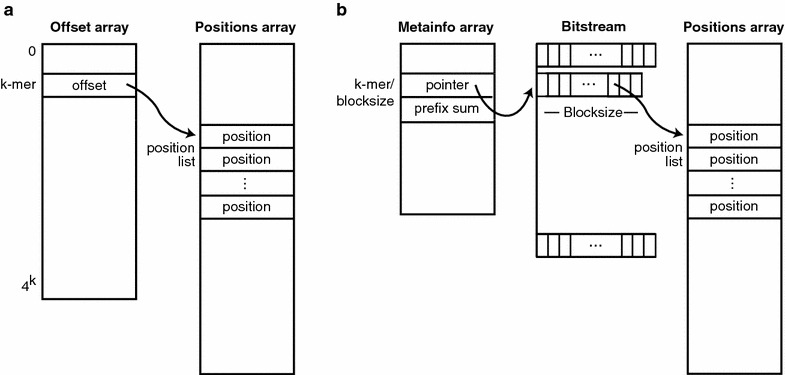
Hash table representation. **a** Standard representation using an offset array that indicates the start of genomic positions for a given *k*-mer. **b** Compressed representation where the offset array has been replaced by a bitstream and a metainformation array. The bitstream contains differences between offsets that have been compressed by bitpacking them into blocks of a given size. The metainformation array contains a pointer and a prefix sum for every block

In genomics, hash tables are typically implemented as a simple lookup table [8], in which an offset array contains pointers into a positions array, for the universe of possible *k*-mers (Fig. 1a). This straightforward table implementation is feasible because of the fixed and relatively small value of *k* needed for our domain. More general domains, having keys of arbitrary or relatively long length, require a hash function to compute a bucket index. Hash functions raise the possibility of collisions, where different keys map to the same bucket index, which then necessitate potentially complex and time-consuming procedures for handling such collisions.

In contrast, a lookup hash table directly provides a list of positions in the genome containing a given *k*-mer. Obtaining that list requires extracting two adjacent values from the offset array: one representing the starting pointer in the positions file for the given *k*-mer, and one for the following pointer (i.e., for the *k*-mer incremented by 1). The difference between these two offsets represents the number of entries in the positions array for the given *k*-mer.

One limitation of lookup hash tables is that the size of the offset array grows exponentially with *k*. Consequently, existing applications have generally used small *k*-mer sizes, such as 11-mers for blastn and blat, and 12-mers for our initial implementation of gmap. However, larger oligomers are generally preferable for performing search, because their greater specificity can greatly reduce the number of candidate genomic positions that need to be processed. Accordingly, the developers of one hash table-based alignment program FusionMap found that using 14-mers gave a speedup of 2–4 times over using 12-mers for alignment [9].

Although modern computers can access increasingly large amounts of primary random-access memory, or RAM, storage space still remains an issue for storing and using large data structures. As the authors of the PatternHunter II program stated, their development was hampered by the the “large memory requirements for multiple hashtables” [10]. Memory considerations also likely precluded the developers of FusionMap from extending their idea beyond 14-mers, since a hash table for 15-mers would require the offset array to be 4 GB in size.

In this paper, we address this problem by using compression. Compression allows larger amounts of information to be stored within a limited amount of memory. Our proposed compression scheme depends on the observation that values in the offset array are monotonically nondecreasing, because they point to successive locations in the positions array. In other words, each value in the offset array is equal to or greater than the previous one. In this situation, compression can be applied to the differences between adjacent values; such differences are generally small integers, which can be compressed more efficiently than large integers. This type of compression has been termed *differential coding*, as opposed to *direct coding*, where successive values may increase or decrease in value, but the original values themselves tend to be small.

Specifically, we explore an approach to compressing offset arrays using a technique called *vectorized bitpacking*, in which blocks of integers are represented with fewer than the 32 bits that are normally allocated for them, and then accessed and processed in parallel. Various schemes for vectorized bitpacking have been developed, but they have been designed for the problem of decoding streams of integers serially. In particular, a recently published scheme for vectorized bitpacking called BP128 [11] is able to achieve very high throughputs for serial decoding, at a rate of 2 billion integers per second, which represents the fastest known method to date.

However, for the genomic problem of compressing and decoding offset arrays in hash tables, we do not require decoding of all integers serially, but rather decoding of individual integers with *random access*. In particular, for a given *k*-mer, we wish to decode two adjacent elements from an arbitrary location within a compressed offset array. In this paper, we introduce a vectorized bitpacking scheme that is relatively compact and extremely fast for this task, and we demonstrate its effectiveness for genomic applications.

## Methods

### Vectorized bitpacking

As its name suggests, vectorized bitpacking involves two concepts: bitpacking and vectorization. In bitpacking, we attempt to conserve computer storage space by using as few bits as possible for storing each integer. Normally, arrays of integers are represented by using a single 32-bit word for each integer, which can represent positive values from 0 to $2^{32}-1$, or approximately 4 billion. However, in many applications, including ours, integer values (or their differences) have small values that do not need all 32 bits in each word. A bitpacking scheme, therefore, uses fewer than 32 bits for each integer when possible.

Many bitpacking schemes are already well established for compressing integers. For example, one example of bitpacking is a universal code, such as Elias gamma coding [12]. In Elias gamma coding, a positive integer needing *c* bits can be encoded using $(2c-1)$ bits, where the first $(c-1)$ bits are zeroes, used as a codeword to indicate that the following *c* bits contain the actual integer. These leading $(c-1)$ bits are needed in serial decoding to delineate the beginning and end of each integer. Likewise, a similar scheme called Elias delta coding [12] represents an integer by encoding its number of bits *c* with Elias gamma, and appending the $(c-1)$ bits that follow the most significant bit. Another universal code uses sets of Fibonacci numbers to encode an integer [13], and is intended to help with decoding in the presence of noise, because it can recover from a damaged bitstream.

The idea behind the second concept, vectorization, is to design algorithms so that they access and process integers in parallel, rather than individually. One type of vectorization makes use of special computer instructions for processing integers in parallel. These SIMD (single instruction, multiple data) instructions exploit specialized 128-bit and 256-bit registers that have been incorporated into processors over the past decade, and which are scheduled to expand to 512-bit registers in the near future. For example, a 128-bit register allows a processor to process four 32-bit integers in parallel, with SIMD operations such as shifting all four integers rightward by a certain number of bits, or adding the four integers in one register to the four integers in another register simultaneously.

Vector-based operations require that integers be encoded with a uniform bit length. Therefore, unlike universal codes, where each integer can have a distinct number of bits, a vectorized bitpacking scheme must allocate the same number of bits for the integers. This does not mean that a single bit width needs to hold for the entire array of integers. Rather, an array can be divided into blocks of a predetermined size, such as 128 or 64 integers at a time, and all integers within each block can then be represented using a uniform number of bits. The bit width for each block is selected to be sufficient for the largest value in that block.

The bit width for each block represents an attribute that needs to be stored in a separate data structure we call the *metainformation* array (Fig. 1b). For random-access applications, the metainformation array also needs to contain, for each block, a pointer into the bitstream where that block begins. If the bit width can be inferred from the total size of the block, then the metainformation array can represent the bit width implicitly based on the difference between successive pointer values.

Differential coding, which computes differences between adjacent integers before encoding, requires computation of a cumulative sum after decoding to obtain the original ascending values. This value is called a *prefix sum*. For serial applications, a prefix sum can be computed as a running total from the beginning of the data stream. However, for random-access applications, it would be computationally expensive to repeatedly compute each prefix sum from the beginning of the data stream. Therefore, computational efficiency can be achieved by storing intermediate prefix sums, one for the start of each block, so that the cumulative sum for any position can be obtained by summing only a relatively few difference values starting from the preceding intermediate prefix sum.

### Layouts for vectorized bitpacking

**Fig. 2:**
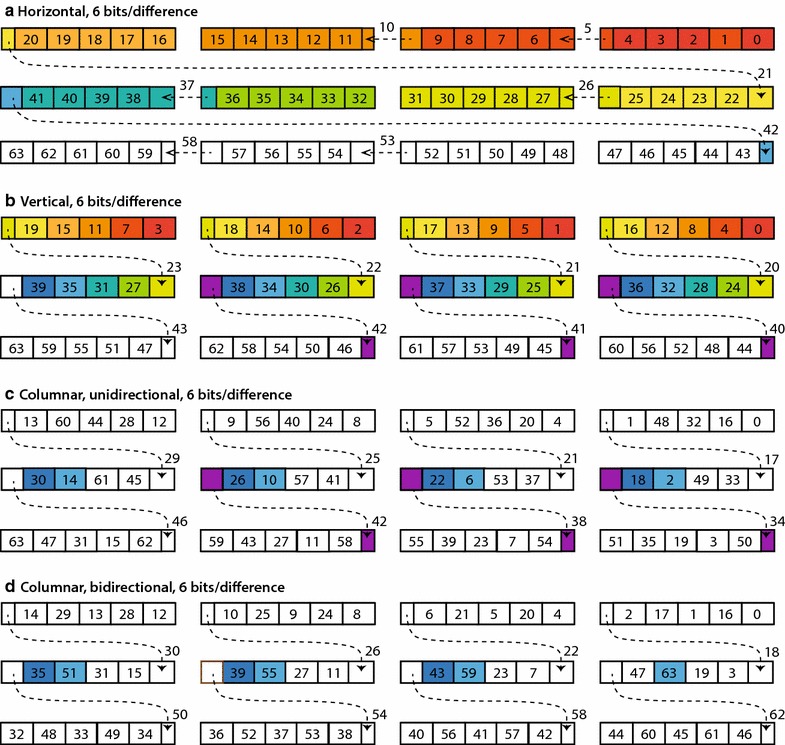
Packing layouts for block-based differential coding. Each layout encodes a block of 64 difference values using 6 bits for each value. Values are placed into three 128-bit registers, each containing four 32-bit words. Each *small box* shows a 6-bit quantity, labeled with an integer *r* that indicates the place for storing the difference value $d_r$. Some 6-bit quantities are spread over two words, as shown by *dashed lines*. Blocks of *color* indicate parallel processing of the difference values needed to decode the value $x_{43}$. **a** *Horizontal* layout, with values stored in index order. **b** *Vertical* layout, with values striped across each set of words, as used in BP128. **c** Columnar layout, unidirectional scheme, with indices at increments of 4 striped across each set of words. **d** Columnar layout, bidirectional scheme

When the bit width for a block is less than 32, more than one integer can be stored in each 32-bit word. The way in which integers are arranged in words can be considered a *layout*. Two layouts have been proposed so far for vectorized bitpacking. In a horizontal layout, adjacent integers are packed next to each other [14]. Figure 2a shows the horizontal layout for a bit width of 6, where each row represents a 128-bit vector of four 32-bit words that are accessed in parallel. Integers that are processed in parallel are shaded with the same color. In the decoding process, this layout requires a separate shift operation for each integer in the word. Although SIMD operations exist for performing distinct shifts for the words in a register, they entail a fair amount of complexity in the decoding algorithm.

**Fig. 3:**
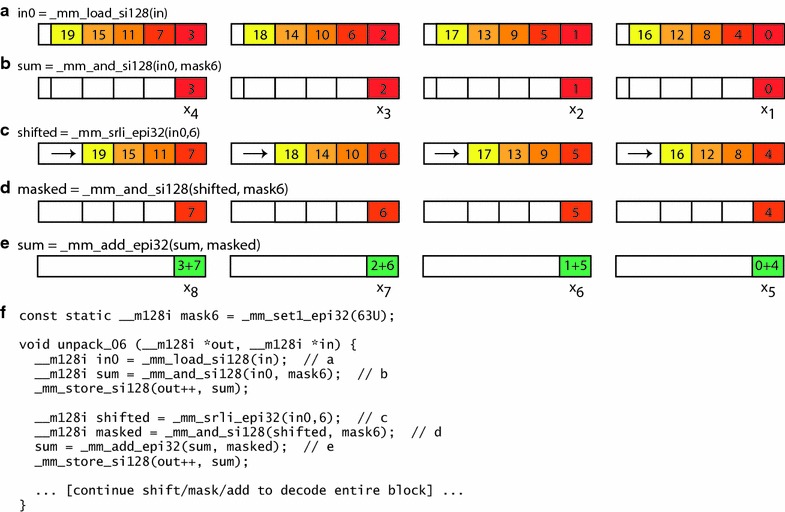
Decoding of vertical layout. An example is shown for the first two cycles of serial decoding of the vertical layout from a block packed with a bit width of 6. *Shaded* regions correspond to the values in Fig. 2b. Source code is shown in part (**f**), with key steps shown graphically in parts (**a**) through (**e**). **a** Loading of the first 128-bit vector from the block. **b** Masking of the first four difference values from the vector. **c**, **d** Shifting and masking of the second four difference values from the vector. **e** Parallel addition of the first and second vectors of difference values. **f** Source code in the C language. *Comments* in the source code correspond to the steps labeled **a** through **e**

In contrast, the vertical layout used in BP128 can be processed by SIMD operations in a more straightforward manner. This layout stripes integers in sets of four across each set of four words [11, 15, 16]. Figure 2b shows a vertical layout for a bit width of 6. Decoding in this layout loads four 32-bit words in parallel (Fig. 3a) and masks their low-order bits to obtain the first four values (Fig. 3b). Then, the next four values can be obtained by right-shifting the words in parallel by the uniform bit width for the block (Fig. 3c), and again masking their low-order bits (Fig. 3d). Since these values represent the differences between the original values, we use a SIMD operation to keep a running sum of the shifted-and-masked low-order bits (Fig. 3e). The remaining values in the block can be decoded using the same SIMD shift and mask steps, with a new set of four 32-bit words loaded whenever all of the currently loaded difference values have been processed. Source code for decoding a vertical layout is shown in Fig. 3f.

**Fig. 4:**
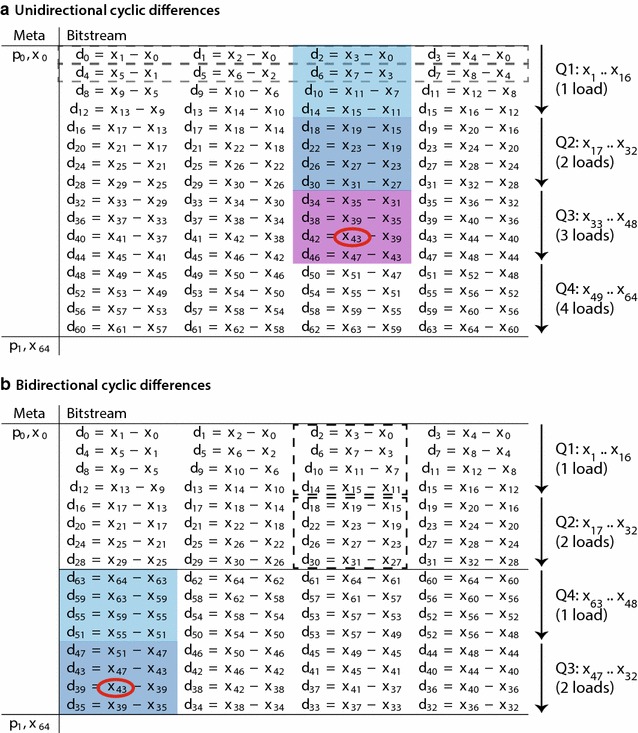
Difference schemes for vectorized differential coding. These schemes show the bitstream and the metainformation values for a given block, with $p_{0}$ and $p_{1}$ indicating the positions in the bitstream where this block and the next block begin, and $x_{0}$ and $x_{64}$ indicating the prefix sum for the two blocks. These schemes show how the original ascending values *x* in a block can be converted to difference values *d*. **a** Unidirectional cyclic differences as used in BP128, except shown here for a block size of 64. The difference $d_i$ involves the original values $x_{i+1}$ and $x_{i-3}$. An exception holds for the *first row*, where differences are taken relative to $x_0$, the prefix sum for the block. *Dashed boxes* indicate the first two processing steps for the vertical layout. **b** Bidirectional cyclic difference scheme, which matches the unidirectional scheme for the first half of the block, but computes differences relative to $x_{64}$ for the second half of the block. *Dashed boxes* indicate the first two processing steps for column 2. In both parts, *shaded regions* correspond to the values needed to compute the *circled values* $x_{43}$, requiring 3 loads for the unidirectional scheme and 2 loads for the bidirectional scheme. The *colors* correspond to those in Fig. 2c, d. *Q1*–*Q4* indicate quarter blocks, and are annotated with the total number of SIMD loads required

In the BP128 algorithm, because four cumulative sums are maintained in parallel, it is not the differences between adjacent values (i.e., $x_{i+1}-x_i$) that we want for differential coding, but rather the differences between each value and the one four elements away (i.e., $x_{i+4}-x_i$). An exception holds for the first four differences in each block, which are computed for $x_1$, $x_2$, $x_3$, and $x_4$ relative to the prefix sum $x_0$ for the beginning of the block, which acts as the starting point for computing the four cumulative sums. The overall cyclic difference scheme for a block of 64 is shown in Fig. 4a.

### Bitpacking for fast random data access

Computing cumulative sums in BP128 is expensive for random-access applications, since the decoding procedure is designed to extract the entire block of 128 integers. In other words, BP128 performs computations that are irrelevant if we want only a single cumulative sum from a given block. To improve vectorized bitpacking for random-access applications, we introduce the following modifications: (1) reduce the block size, (2) arrange integers within each block in a columnar layout, and (3) utilize a bidirectional scheme for computing differences between values. These modifications make it possible to decode a single value in a differentially encoded block with greater efficiency than the BP128 scheme. We discuss each of these modifications in turn.

### Reduced block size

In vectorized bitpacking, integers are encoded using a uniform bit width for each block. The BP128 scheme is so named because it packs integers in blocks of 128. One reason for using that block size is that encoding 128 integers, regardless of which bit width from 0 through 32 is used, results in a bitstream that is aligned on a 128-bit word boundary, which can improve the speed of SIMD load instructions on some computer systems [17].

For serial applications, since all values in a block are decoded, the choice of block size should have little effect on overall decoding speed. In fact, a larger block size may be slightly more efficient for exhaustive decoding, because each call to a decoding procedure for a single block can handle a larger amount of work.

However, for random-access applications, a smaller block size is potentially advantageous in reducing the amount of computation needed within a given block to obtain a single value. In other words, a block size of 64 should roughly halve the number of SIMD load, shift, mask, and addition operations needed to decode a single value, relative to a block size of 128. The actual effect on decoding speed may not achieve these theoretical savings, though, because of the time required for memory access and the effect of pipelining of operations by the processor. The drawback of halving the block size is that twice as many pieces of metainformation (pointers and block prefix sums) are required, thereby increasing the amount of space needed.

One advantage of a block size of 64 is that it allows us to restrict the uniform bit widths to even values. As we mentioned previously, the BP128 scheme aligns its block of 128 integers at a 128-bit word boundary, for every bit width from 0 through 32, without any need for zero padding. For example, for a bit width of 3, a block of 128 integers requires exactly three 128-bit registers for storage. However, for a block size of 64, odd bit widths do not offer any savings in storage relative to the next larger even bit width. For this block size, a bit width of 3 uses one and a half 128-bit registers, effectively the same space as for a bit width of 4, which uses two full 128-bit registers. Furthermore, the decoding procedures for odd bit widths are more complex than those for even bit widths, because odd sizes do not fit evenly into 32-bit registers, necessitating more SIMD operations for merging integers that cross from the end of one register into the beginning of the next one (as indicated by the dashed lines in Fig. 2). Consequently, decoding speeds for even bit widths have been shown to be slightly faster than for odd bit widths [16].

Although we will continue our exposition of our bitpacking scheme using a block size of 64, we will consider experimentally an even smaller block size of 32. Such a block size would allow us to restrict bit widths to multiples of 4, and should theoretically reduce the number of SIMD operations further, albeit at a cost of further doubling the storage needed for metainformation. A block size of 64 is somewhat more natural for genomic applications, since it is a power of 4, meaning that the beginning of each block can be associated with a specific *k*-mer. However, a natural interpretation is not essential for either serial or random-access decoding, so arbitrary block sizes can be considered, although some may turn out to be more or less optimal for compression or speed.

### Columnar packing layout

We can greatly improve the efficiency of random access for differential decoding by noting that most of the SIMD computations in a BP128 decoding are wasted if we need only a single cumulative sum in the block. For example, consider the case where we require only the cumulative sum for the 43rd entry, or $x_{43}$. Fig. 2a, b show with color coding the loads needed for the horizontal and vertical layouts. In both layouts, all difference values from $d_0$ through $d_{42}$ must be decoded and summed in order to obtain the value for $x_{43}$. For the vertical layout, a total of 11 sets of decoding steps are needed to reach $x_{43}$, with the first two decoding steps represented by dashed boxes in Fig. 4a.

However, the critical path for obtaining $x_{43}$ can be shortened if we can load the difference values by columns instead, as shown by the light blue, dark blue, and violet shading in Fig. 4a. Then, the path to computing $x_{43}$ involves summing the difference values in the columnar path from $d_{2}$ through $d_{42}$, and requires only three decoding steps instead of 11.

To decode difference values by columns instead of rows, we propose a new packing layout called a columnar layout (Fig. 2c). In this layout, we pack one column at a time, with the 16 entries in column 0 ($d_{0}$, $d_{4}$, ..., $d_{60}$) striped in groups of four across available spaces in a series of 128-bit vectors. The remainder of the block is similarly packed in column order for columns 1, 2, and 3. The SIMD loads needed for obtaining $x_{43}$ are shaded in light blue, dark blue, and violet in Fig. 2c.

### Bidirectional difference scheme

The columnar layout requires us to implement a separate decoding procedure for each column in each quarter-block, where the first quarter-block (Q1) contains block positions 1–16; Q2 contains 17–32; the Q3 contains 33–48; and Q4 contains 48–63 (Fig. 4a). Entries in Q1 need a single SIMD load of integers, whereas entries in Q2, Q3, and Q4 need two, three, and four SIMD loads, respectively.

In this scheme, the Q3 and Q4 entries require more loads, since those values are farther away from the prefix sum $x_0$, which constitutes the starting point for computing cumulative sums. However, we can improve the situation if we consider that each block has both a beginning prefix sum $x_{0}$ and an ending prefix sum $x_{64}$, which is already stored in the metainformation array as the beginning prefix sum for the next block. Therefore, we can compute the differences for the first half-block (values $x_{0}$ through $x_{32}$) relative to the beginning prefix sum, and the differences for the second half-block (values $x_{32}$ through $x_{64}$) relative to the ending prefix sum. (The sum $x_{32}$ can be computed from either the beginning or the ending prefix sum.) Hence, to compute a value for the second half of the block, we can perform SIMD operations just on that half, and then subtract the resulting cumulative sum from $x_{64}$.

This idea gives rise to a bidirectional difference scheme (Fig. 4b), rather than the unidirectional scheme (Fig. 4a) that we have been considering so far. Consequently, the bidirectional scheme allows for fewer loads of data for quarter blocks Q3 and Q4, which now require only two and one SIMD loads, respectively. For any given entry $x_{r}$ at block position *r*, we can compute the desired column by first computing the distance $\delta = 31 - |r - 32|$ to the nearest prefix sum, which is equivalent to $\delta = r - 1$ for $r\le 32$ and $\delta = 63 - r$ for $r\ge 32$. Then we require only the values in column ($\delta \bmod 4$), beginning from row 0 in the half-block to row $\lfloor \delta /4\rfloor$.

For our example of computing $x_{43}$ in Q3, the desired column in the bidirectional scheme is shaded in Fig. 4b as light blue and dark blue, requiring two SIMD loads, as opposed to three. These shades correspond to the bidirectional columnar layout shown in Fig. 2d. In this layout, we pack the integers in the first half-block according to the columns in Fig. 4b, and then the integers in the second half-block, again according to columns, but in reverse order.

### Within-column summation

To decode a value from the bitcompressed differences, we need a total of 256 specialized procedures, to handle all combinations of the 16 possible even bit widths, with four columns for each of the four quarter-blocks. A jump table for $x_{r}$ can be used to invoke the appropriate decoding procedure, based on (1) the bit width, (2) the column ($\delta \bmod 4$), and (3) the quarter-block, based on $\lfloor r/16\rfloor$. A bit width of 0 indicates the special case in which all values in the block are zero; for this case, the decoding procedure can simply return the prefix sum $x_{0}$ for any entry in the block.

**Fig. 5:**
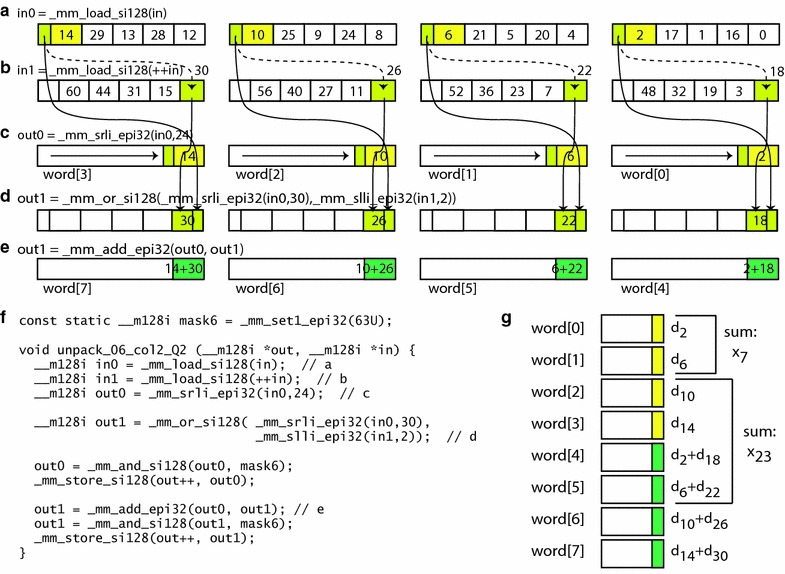
Decoding of columnar layout. An example is shown for decoding column 2 for quarter block Q2 from a block packed with a bit width of 6. Source code is listed in part (**f**), with key steps shown graphically in parts (**a**) through (**e**), and a final summation step in part (**g**). **a**, **b** Loading of two 128-bit vectors from the block. **c** Parallel (SIMD) right shift of each 32-bit word by 24 bits, to move $d_2$, $d_6$, $d_{10}$, and $d_{14}$ into the lowest 6 bits. **d** Recombining of $d_{18}$, $d_{22}$, $d_{26}$, and $d_{30}$, which are split between two 128-bit vectors in the block, using a parallel right shift of the first vector by 30 bits and a parallel left shift of the second vector by 2 bits. **e** Parallel addition of the first and second vector of differences. **f** Source code in the C language. *Comments* in the source correspond to the steps labeled **a** through **e**. **g** Difference results shown as an array of 32-bit words. The value $x_7$ can be obtained by adding two terms from the array, while the value $x_{23}$ can be obtained by adding four terms

As an example of one of these 256 procedures, Fig. 5 shows a procedure for decoding column 2 for the Q2 quarter block from a block with a bit width of 6. The two decoding steps are also shown by the dashed boxes in Fig. 4b. The first decoding step yields four difference values, shown as words 0 through 3 in Fig. 5c, and the second decoding step yields another four difference values, shown in Fig. 5d. Our procedure then performs a final SIMD addition to add the first set of difference values to the second set, thereby yielding words 4 through 7 in Fig. 5e. Words 0 through 7 from Fig. 5c, e can be stored in an array, as shown in Fig. 5g.

Through appropriate summations of the first four words, we can obtain the cumulative sums $x_{3}$, $x_{7}$, $x_{11}$, and $x_{15}$ for Q1, by using 1, 2, 3, and 4 terms, respectively. It would therefore seem, by extension, that obtaining the cumulative sums $x_{19}$, $x_{23}$, $x_{27}$, and $x_{31}$ for Q2 would require computing over 5, 6, 7, and 8 terms, respectively. However, our final SIMD computation is designed to perform some additions in parallel, and therefore makes summation of at most four terms sufficient in all cases. For example, to compute $x_{23}$, we need add only words 2 through 5, which represent the four quantities $d_{10}$, $d_{14}$, $(d_{2}+d_{18})$, and $(d_{6}+d_{22})$ instead of the expected addition of six separate quantities $d_{2}$, $d_{6}$, $d_{10}$, $d_{14}$, $d_{18}$, and $d_{22}$.

From this example, we also see that the entries for Q1 are decoded on the way to decoding the Q2 entries. Likewise, the entries for Q4 are decoded on the way to decoding the Q3 entries. Therefore, we need to devise only 128 distinct procedures for decoding the Q2 and Q3 quarter-blocks, with the Q1 and Q4 procedures being derived easily as the initial parts of those procedures.

### Retrieval of two adjacent offset values

Although some applications of differential coding require retrieval of only a single value, our domain of interest, genomic alignment, requires us to decode two adjacent offsets. These two values specify the endpoints for the list of genomic positions for a given *k*-mer, representing the start and end of the list in the positions array. In other words, when we decode $x_{23}$ to obtain the start of the list, we must also decode $x_{24}$ to obtain the end of the list. For a vertical layout, the additional value requires just one more difference value to be extracted and added. However, since our bitpacking scheme is designed to extract columns of values, it requires an entire second decoding procedure, in this case to extract column 3 of the Q2 entries. The most straightforward implementation to retrieve two adjacent offset values would be to make separate calls to two decoding procedures, which we call a *two-pass* implementation.

However, a slight improvement can be made by noting that in our difference scheme, adjacent values will be in neighboring columns (where we consider column 3 and column 0 to be neighboring). Therefore, decoding of column 0 will always be followed by column 1; column 1 by column 2; column 2 by column 3; and column 3 by column 0. Because adjacent columns are packed in adjacent stripes in the columnar layout, difference values from the second column may have already been loaded during computations for the first column. We can therefore combine the two separate decoding procedures to yield a *one-pass* implementation. A one-pass implementation can achieve some efficiency savings by avoiding duplicate SIMD loads of registers. These one-pass procedures can be derived by appropriate merging of individual two-pass procedures, taking advantage of registers that have already been loaded.

## Evaluation

### Experimental setup

We evaluated the tradeoff between space and time for various methods to retrieve offsets from a hash table, both for a single offset and a pair of adjacent offsets. We used genomes of different sizes, namely, the fly genome (D. melanogaster version 5.25.64), chicken genome (Gallus gallus version 4), and human genome (version hg19).

We implemented our methods within the Succinct Data Structure Library (SDSL) 2.0 package, which is publicly available as C++ source code [18]. Our bitpacking code derives from a revision [16] of the original BP128 work [11], which we modified to implement our columnar method. To be consistent with the naming of classes in the SDSL package, our classes are named bp64_encv_vector for the BP64-vertical layout; bp64_encc_vector for BP64-columnar; and bp32_encv_vector for BP32-columnar. We also implemented benchmarking procedures to compare our methods with existing compression methods in SDSL, namely, Elias gamma, Elias delta, and Fibonacci encoding, all with a block size of 64, as well as with an uncompressed int_vector method available in SDSL. We added versions of these compression methods that encode the incremental value of 0 efficiently, by subtracting 1 from each positive integer during encoding and adding an equivalent amount when decoding an integer within a block. These modifications were implemented with the assistance of the author of SDSL (personal communication). Without these modifications, execution times of the original Elias and Fibonacci methods were extremely slow in our early benchmarking experiments.

All timing experiments were performed on a reserved Linux computer having 32 Intel Xeon E5-2667 v3 8-core processors running at 3.20 GHz. The computer had total memory of 264 GB and cache memory of 20 MB. The SDSL 2.0 library was compiled with the GNU g++ compiler, version 4.9.0, with the default settings, which turned off debugging code, and added the compiler flags “-O3 -ffast-math -funroll-loops -msse4.2”.

Our benchmarking code generates 10 million random values uniformly over the space of possible queries and measures the average time to retrieve results for each query. Data structures were either read into memory from the filesystem or generated de novo in memory from the input files. A checksum was computed over the results to ensure that the methods gave consistent results and that the compiler did not optimize out the query. All timing measurements were repeated for 9 trials, with each trial involving different random values generated and testing different compression strategies in a randomly selected order. Results are summarized by the median over the 9 trials. We also measured the time for iterating through the 10 million queries, obtaining the test offset index, and performing the checksum, and subtracted the median times from all runs. These times amounted to a negligible fraction of the overall running times.

Source code for all bitpacking implementations of hash tables (and our companion research on bitpacking for enhanced suffix arrays) is made available as Additional file 1. The package is a modification of SDSL 2.0 that includes our new methods, benchmarking code, and alteration of existing methods to encode the incremental value of 0 efficiently. Genomic input files for the benchmarking experiments are hosted on a public Web site, with downloading instructions available within the package. Alternatively, we have prepared a package, available for download as Additional file 2, that allows users to generate their own benchmarks from any DNA or RNA source.

### Retrieving a single offset value

We generated genomic hash tables using 15-mers, sampled every 3 bp in the genome. We used the offset array from each hash table as a source of monotonically nondecreasing values to be compressed in the following ways: (1) uncompressed, using the SDSL int_vector method, in which each offset was represented as a 4-byte quantity; (2) BP64-vertical, which is identical to the BP128 format as proposed by [11] that uses a unidirectional difference scheme and vertical cyclic packing format, but with a block size of 64 values and using only even-valued bit widths; (3) BP64-columnar, as proposed in this paper, using a bidirectional difference scheme and columnar packing format; (4) BP32-columnar, also proposed here, with a block size of 32; (5) Elias gamma encoding, with a block size of 64 values, as implemented in SDSL; (6) Elias delta encoding, with a block size of 64 values, as implemented in SDSL; and (7) Fibonacci encoding, with a block size of 64 values, as implemented in SDSL.

**Fig. 6:**
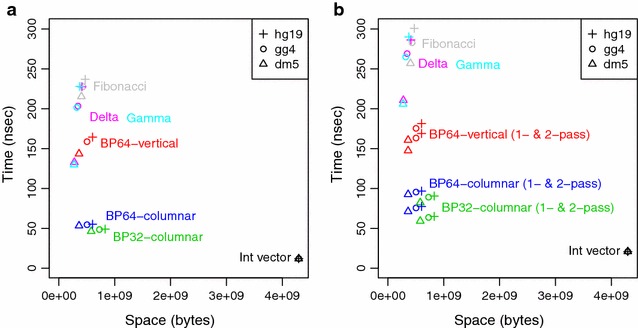
Results for retrieval of offset array values. Space and time usage for retrieving offset array values under various compression schemes. Offsets are obtained from 15-mer hash tables for the fly (dm5), chicken (gg4), and human (hg19) genomes. *Graphs* show the retrieval time in nanoseconds per query as a function of the space required in bytes for each format. **a** Retrieval results for a single value. **b** Retrieval results for two adjacent values. Methods tested: storage as 32-bit integers (Int vector), or compressed using Elias gamma coding (Gamma), Elias delta (Delta), Fibonacci, BP64-vertical format, or the BP64-columnar and BP32-columnar schemes introduced here. For retrieving two adjacent values using vectorized bitpacking formats, results are shown for both two-pass (slower) and one-pass (faster) implementations

The results for retrieving a single offset value are shown in Fig. 6a. These results show that retrieval time is largely independent of genome size, which derives from the fact that the offsets file length depends instead on the *k*-mer size. The uncompressed format gives the fastest times at 12 ns/query, but requires 4 GB of space ($4^{15}$ entries with 4 bytes required per entry). The Elias gamma, Elias delta, and Fibonacci formats produce compact representations that are 6–11 % of the uncompressed format, but generally have the slowest retrieval times, with over 130 ns/query for the fly genome, over 200 ns/query for the chicken genome, and 228–237 ns/query for human genomes. The BP64-vertical format requires slightly more space, at 8–14 % of the uncompressed format, but is 1.3–1.4 times faster than the SDSL methods, except for the fly genome, where the Elias gamma and delta methods are 10 % faster than the BP64-vertical method. The BP64-columnar format requires the same amount of space as BP64-vertical, but has retrieval times that are 2.7–3.0 times faster. The BP32-columnar format requires the most space among the bitpacking routines we tested, at 13–19 % of the uncompressed format (or 38–60 % more space than BP64-columnar), with times that are 12–15 % faster than BP64-columnar.

### Retrieving two offset values

The results for retrieving two adjacent values are shown in Fig. 6b. The space measurements remain the same for each format relative to the first experiment, and only the time measurements differ. For the uncompressed, Elias gamma, Elias delta, and Fibonacci formats, we made two separate calls to the retrieval function. When we compare the results of Fig. 6b with those of Fig. 6a, we observe that times for two calls for the uncompressed data and SDSL methods were 1.2–1.7 times those for a single call. The fact that the retrieval time did not double reflects the effects of memory caching. For the BP64-vertical format, two separate calls to retrieve adjacent values required 1.1x of the time for a single call for one value, whereas for the BP64-columnar and BP32-columnar formats, the extra time required was a factor of 1.7-1.8. The difference between the vertical and columnar results suggests that memory caching is more effective for the vertical format. This makes sense, because for the vertical format, the next adjacent value is likely to be decoded in the same group of four integers as the first value. But for the columnar format, an adjacent value requires another column of 128-bit registers to potentially be loaded and to be decoded.

For the vectorized bitpacking formats, we implemented one-pass methods, which retrieve two adjacent offset values by combining memory retrievals whenever possible.  These methods can be contrasted with the two-pass approach that uses two separate calls to retrieve the desired values. As shown in Fig. 6b, the one-pass methods have a greater effect for the columnar formats, with the vertical format showing speedup by a factor of 1.07, but the columnar formats showing a speedup of 1.2–1.4. Overall, the one-pass methods for the BP64-columnar format are 2.1–2.2 times faster than the one-pass method for BP64-vertical, and the one-pass methods for the BP32-columnar format are 2.5–2.6 times faster. The comparison with the Elias gamma, Elias delta, and Fibonacci coding methods show a speedup of 2.9–3.9 times for BP64-columnar and 3.5–4.6 times for BP32-columnar, with the greater speedup seen for the chicken and human genomes. However, we should note that the SDSL methods were tested as two-pass retrievals, since no alternative one-pass implementations are available in the package.

## Discussion

Our research in this area has been motivated largely by attempts to improve the space and time efficiency of our genomic alignment programs gmap [7] and gsnap [19]. Initially, both programs used a hash table of 12-mers. We subsequently increased the *k*-mer size to 15-mers, by compressing the hash table using an Elias gamma compression scheme (September 2011 release), which improved the speed of gsnap by a factor of approximately 6–8. We then changed the bitpacking scheme to the BP64-vertical format and added a suffix array algorithm (October 2013 release), which gave an additional speedup of 4–7 times, depending on the similarity between the reads and the genome. The research in this paper represents our subsequent studies to improve the decoding speed of bitpacked data for random access, which we have used to further increase the speed of our programs with a BP64-columnar format (April 2014 release).

Our study leads to some insights for practical genomic implementations. For differential coding of offset arrays in hash tables, our BP64-columnar and BP32-columnar formats offer significant improvements in random-access decoding speed relative to the fastest known methods to date, with a threefold increase in speed for retrieving a single value and a twofold increase for two adjacent values. Between the two columnar alternatives, BP32-columnar gives a 12 % increase in speed for retrieving one value and a 20 % increase in speed for retrieving two adjacent values, but requires 40–60 % more space. Therefore, BP64-columnar appears to give a better balance between storage space and decoding speed, which underlies our decision to use this representation for our alignment programs.

Apart from this study, compression of small integers has not been widely applied or studied for genomic alignment. Some researchers [20] have used integer codes to compress the short reads themselves, after they have been aligned to a genome. Another application of integer codes has been to compress genomes or other nucleotide sequence datasets, either by themselves [21] or as differences relative to another genome [22].

In particular, although much research effort has gone into developing effective techniques for compressing genomic data structures [23, 24], little work has been done on compression techniques for hash tables, despite their prevalence in many bioinformatics applications. Hash tables are closely related to inverted index lists, which are used widely for indexing text and Web pages for rapid search [25], and where compression techniques have been applied [26]. However, those systems do not exploit the fact that a genomic hash table indexes an exhaustive set of *k*-mers, which presents domain-specific opportunities for compression.

An early system called cafe [27] for aligning sequences to databases did use compression techniques in representing a genomic index. However, that system used an inverted index structure rather than a hash table, so it lacked an exhaustive set of offsets. Instead, it used integer coding to compress differences in the position table, achieving compression of 4–6 times smaller than the uncompressed size. We believe that compression of offsets is much more efficient than compressing positions, since for large genomes adjacent positions do not have the same pattern of closeness that offsets do and therefore do not compress as well. Moreover, to achieve maximal speed in combining hash table lookups, in procedures like the spanning set or complete set algorithms in gsnap [19], it is faster to have lists of positions available in an uncompressed format.

One contribution of this paper has been to show how SIMD operations can be applied to genomic representations. Vectorization has found applications in other areas of bioinformatics, such as speeding up Smith-Waterman dynamic programming for nucleotide alignment [28–30] and profile HMM searches [31]. Likewise, we have applied vectorization to other parts of our alignment programs, such as computing mismatches between a query sequence and a genomic segment; constructing a localized hash table for a genomic region; performing dynamic programming alignment at the nucleotide level; and filtering results from a list of genomic positions to satisfy a given range of coordinates [32].

The BP64-columnar scheme in this paper is a solution to the general problem of differential coding with random access, and complements the BP128-vertical scheme for serial access. Therefore, our scheme conld be considered for any problem where a monotonically nondecreasing set of integers can be compressed and then retrieved by random access. In particular, many bitpacking schemes have been proposed for text retrieval [33], and database tables often have a format similar to our offset array. Applying our methodology to other domains is beyond the scope of this paper. However, by providing our methods in a standard package, we hope to facilitate further work by researchers to experiment with our methods and to use them more widely.

## Additional files

10.1007/s13015-016-0069-5 Source code. Source code in an archive format, using tar and bzip2, for all bitpacking implementations of hash tables, based on a modification of the SDSL 2.0 package. Package also includes the benchmarking programs used for timing experiments. This is the same package as provided in our companion paper on bitpacking for enhanced suffix arrays.

10.1007/s13015-016-0069-5 Source code for constructing benchmarks. Source code in an archive format, using tar and bzip2, for users to generate their own benchmarks for any genomic text in FASTA format. This is the same package as provided in our companion paper on bitpacking for enhanced suffix arrays.
