# Supplementary material for: Bitpacking techniques for indexing genomes: I. Hash tables
Source: Algorithms Mol Biol. 2016 Apr 18;11:5. doi: 10.1186/s13015-016-0069-5 (PMC4835851; doi:10.1186/s13015-016-0069-5)
Supplement: Supplementary file 1 — 10.1007/s13015-016-0069-5 Source code. Source code in an archive format, using tar and bzip2, for all bitpacking implementations of hash tables, based on a modification of the SDSL 2.0 package. Package also includes the benchmarking programs used for timing experiments. This is the same package as provided in our companion paper on bitpacking for enhanced suffix arrays. [file 13015_2016_69_MOESM1_ESM.bz2 › sdsl-lite-modified/extras/cheatsheet/viz.pdf]

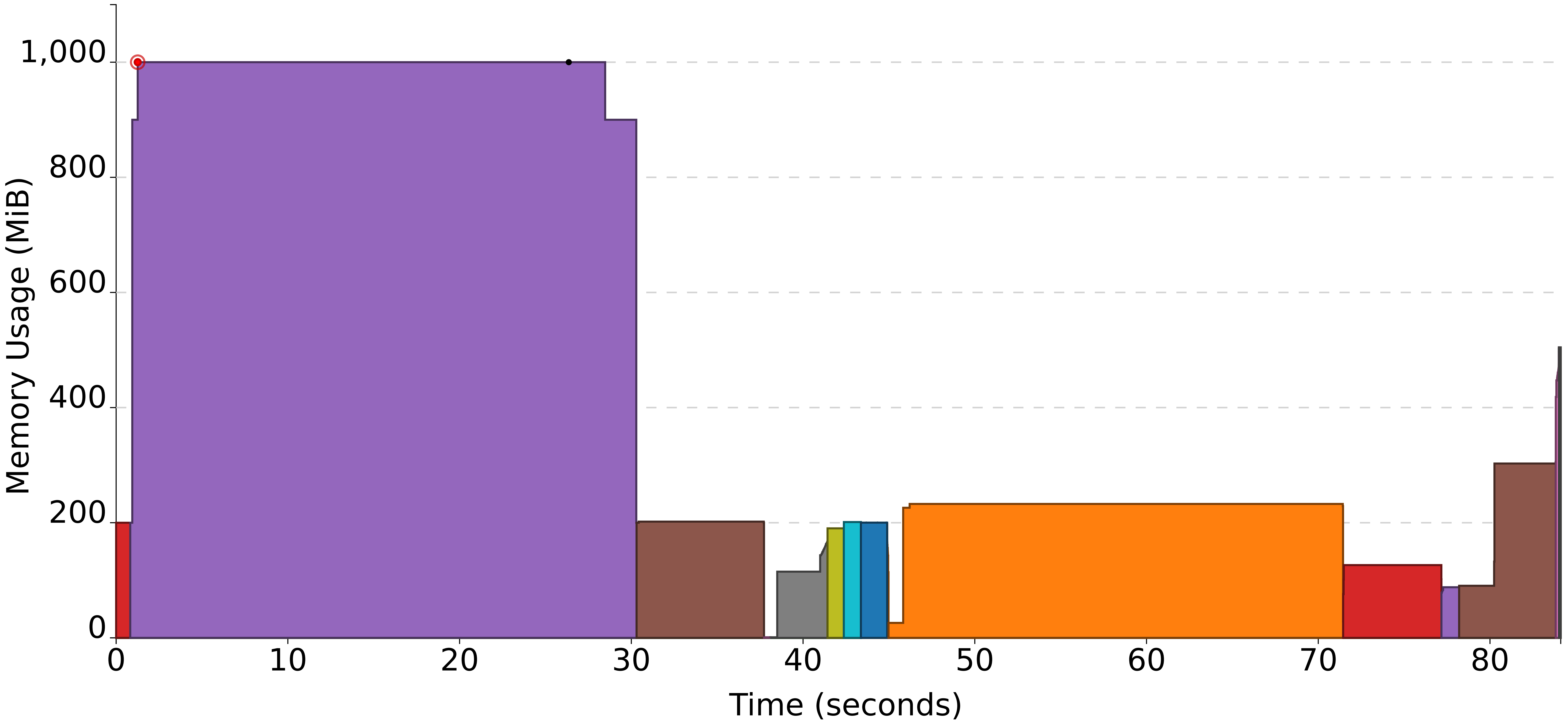

Memory Usage (MiB)
1,000
800
600
400
200
0
0
10
20
30
40
50
60
70
80
Time (seconds)
